# Atomistic Molecular Dynamics Simulations of the Initial Crystallization Stage in an SWCNT-Polyetherimide Nanocomposite

**DOI:** 10.3390/polym9100548

**Published:** 2017-10-24

**Authors:** Victor M. Nazarychev, Sergey V. Larin, Alexey V. Lyulin, Theo Dingemans, Jose M. Kenny, Sergey V. Lyulin

**Affiliations:** 1 Institute of Macromolecular Compounds, Russian Academy of Sciences, Bol’shoi pr. 31 (V.O.), St. Petersburg 199004, Russia; nazarychev@gmail.com (V.M.N.); selarin@macro.ru (S.V.L.); jose.kenny@unipg.it (J.M.K.); 2 Theory of Polymers and Soft Matter Group, Technische Universiteit Eindhoven, P.O. Box 513, 5600 MB Eindhoven, The Netherlands; a.v.lyulin@tue.nl; 3 Department of Applied Physical Sciences, University of North Carolina at Chapel Hill, Murray Hall 1113, 121 South Road, Chapel Hill, NC 27599-3050, USA; tjdatunc@email.unc.edu; 4 Materials Science and Technology Centre, University of Perugia, Loc. Pentima, 4, 05100 Terni, Italy

**Keywords:** polyetherimides, crystallization, molecular dynamics, nanocomposite, π–π interactions

## Abstract

Crystallization of all-aromatic heterocyclic polymers typically results in an improvement of their thermo-mechanical properties. Nucleation agents may be used to promote crystallization, and it is well known that the incorporation of nanoparticles, and in particular carbon-based nanofillers, may induce or accelerate crystallization through nucleation. The present study addresses the structural properties of polyetherimide-based nanocomposites and the initial stages of polyetherimide crystallization as a result of single-walled carbon nanotube (SWCNT) incorporation. We selected two amorphous thermoplastic polyetherimides ODPA-P3 and aBPDA-P3 based on 3,3′,4,4′-oxydiphthalic dianhydride (ODPA), 2,3′,3,4′-biphenyltetracarboxylic dianhydride (aBPDA) and diamine 1,4-*bis*[4-(4-aminophenoxy)phenoxy]benzene (P3) and simulated the onset of crystallization in the presence of SWCNTs using atomistic molecular dynamics. For ODPA-P3, we found that the planar phthalimide and phenylene moieties show pronounced ordering near the CNT (carbon nanotube) surface, which can be regarded as the initial stage of crystallization. We will discuss two possible mechanisms for ODPA-P3 crystallization in the presence of SWCNTs: the spatial confinement caused by the CNTs and π–π interactions at the CNT-polymer matrix interface. Based on our simulation results, we propose that ODPA-P3 crystallization is most likely initiated by favorable π–π interactions between the carbon nanofiller surface and the planar ODPA-P3 phthalimide and phenylene moieties.

## 1. Introduction

Semicrystalline all-aromatic polymers are widely used as high-performance materials in various industries [1,2,3,4,5,6,7,8,9]. However, their mechanical properties cannot compete with metal-based materials, and the addition of reinforcing fibers or nanofillers is currently used to improve the thermo-mechanical properties of polymer matrices. Crystallization of amorphous polymers is one way to improve their mechanical properties. Incorporation of carbon-based nanofillers into the polymer matrix is known to accelerate the crystallization process through nucleation, and in turn, this can lead to an improvement in the polymer mechanical properties [10,11,12,13,14]. In particular, the nucleating ability of carbon nanotubes (CNTs) has been reported by several groups. A wide variety of polymer matrices has been investigated, including polyethylene [15,16,17], polyacrylonitrile [18], polypropylene [12,13,19], nylon [20,21,22] and aromatic polyetherimides (PEIs) [23,24,25].

The nanofiller surface may have an impact on the structure of neighboring polymer chains [26]. When the nanofiller size is comparable to the size of a polymer chain, the nanofiller particles may create a geometric confinement, which is known to result in the orientation of adjacent polymer chains [16]. Furthermore, the nanofiller surface may confine and slow down the segmental relaxation of polymers [27,28]. A significant structural ordering near the nanofiller surface may result via specific interactions at the polymer-nanofiller interface, especially when the size of the nanofiller particle exceeds that of the polymer chains, similar to the size-dependent soft epitaxy [16,26]. The structural properties of the polymer near the nanofiller are also determined by the chemical composition of the participating components [10,29,30,31,32,33,34]. For aliphatic polymers, researchers have shown that there are specific interactions between CH–groups and CNT atoms [35]. In the case of conjugated polymers, there is evidence that due to π–π interactions, chain fragments may orient along the nanofiller surface [36,37].

However, questions of why crystallization of the polymer matrix takes place near the nanofiller surface and what is the role of geometric confinement or other specific interactions (for example, π–π) remain largely unanswered, and this is in particular true for aromatic heterocyclic polymers [38].

A detailed investigation of the nanocomposite structure at the polymer-nanofiller interface allows for a more in-depth understanding of the mechanisms of crystallization initiation of heterocyclic polymers as a result of carbon nanofiller incorporation. This is essential for the development of new nanocomposite materials with improved mechanical properties. Atomistically detailed computer-aided simulations make it possible to study the structure of the polymer-nanofiller interface on the scale of single atoms [39,40,41,42] and, therefore, help to provide insights into the structural ordering near the carbon nanofiller surface and why it improves the nanocomposite mechanical properties.

Simulation studies of the structural properties of polymers near the nanofiller surface have been performed using coarse-grained [43] and atomistic [44] models. Rather simple polymers in terms of chemical structure have been simulated so far, such as polyethylene [43,45] or poly(trimethylene terephthalate) [44], and the effects of single-walled nanotubes (SWCNTs) and graphene on the structural properties of alkane polymer melts have been considered [46]. Researchers have demonstrated that the structural ordering of polymer chains near the CNT surface may be the result of geometric confinements due to the presence of the nanofiller. The graphene surface may have a different impact; according to the results of Yang et al. [46], the polymer-graphene interface structure is determined by specific molecular interactions between the participating components. Simulations have been performed to study the structural ordering of conjugated polymers near the carbon nanofiller surface. Asadinezhad et al. have reported that the planar aromatic rings of poly(trimethylene terephthalate) are oriented parallel with the CNT axis, which may be attributed to π–π interactions [44]. More recent research has focused on fully-aromatic high-performance polymers with high temperature stability, as for example, thermoplastic polyimides [7,47,48]. Typical commercially available thermoplastic PEIs do not crystallize in the presence of nanofillers. However, at the Institute of Macromolecular Compounds of the Russian Academy of Sciences, a crystallizable thermoplastic polyetherimide R-BAPB based on 1,3-*bis*-(3′,4-dicarboxyphenoxy)-benzene (dianhydride R) and 4,4′-*bis*-(4″-aminophenoxy)-diphenyl (diamine BAPB), was synthesized [49], which was used as a polymer binder to produce nanocomposites by melt processing techniques. In our previous studies, we used atomistic molecular dynamics (MD) simulations to investigate the structural ordering of planar moieties of the R-BAPB chain near the surface of SWCNTs [50,51] and graphene [52,53]. It is important to note that the structural ordering of the planar moieties of R-BAPB leads to an improvement in mechanical properties at the polymer-nanofiller interface. Our simulations [52] showed that the average values of the elastic modulus and yield stress of crystallized R-BAPB near the nanofiller surface increased in comparison to R-BAPB in the amorphous state. We were able to clearly demonstrate that the structural ordering of the R-BAPB planar moieties near the nanofiller surface resulted in an improvement in the mechanical properties of the polymer-CNT nanocomposite [52]. The model developed by us previously made it possible to qualitatively describe the initial stages of R-BAPB crystallization near the CNT surface, which was confirmed by experimental results [24,25]. Based on our previous work, we want to stress that special care should be taken to choose the force field, in particular for the parameterization of electrostatic interactions in the case of heterocyclic polyetherimides with polar groups [50,51,52,53,54,55,56,57,58,59,60,61,62].

Quite new is the fact that the incorporation of SWCNTs may nucleate crystallization even in amorphous polyetherimides. In fact, the structural, thermal and mechanical properties of two amorphous thermoplastic polyetherimides ODPA-P3 and aBPDA-P3 based on 3,3′,4,4′-oxydiphthalic dianhydride (ODPA), 2,3′,3,4′-biphenyltetracarboxylic dianhydride (aBPDA) and diamine 1,4-*bis*[4-(4-aminophenoxy)phenoxy]benzene (P3) were investigated [63,64], and the authors showed that incorporation of SWCNTs resulted in the crystallization of the ODPA-P3 matrix, as opposed to aBPDA-P3, which remained amorphous after the addition of the SWCNTs. The mechanism of SWCNT-induced crystallization of ODPA-P3 remains unclear; in fact, it is not clear whether, for example, crystallization is initiated due to spatial confinement, because of the carbon nanofiller presence, or due to π–π interactions between the aromatic cycles of the CNTs and the PEI phenylene and/or heterocyclic (phthalimide) moieties.

**Figure 1:**
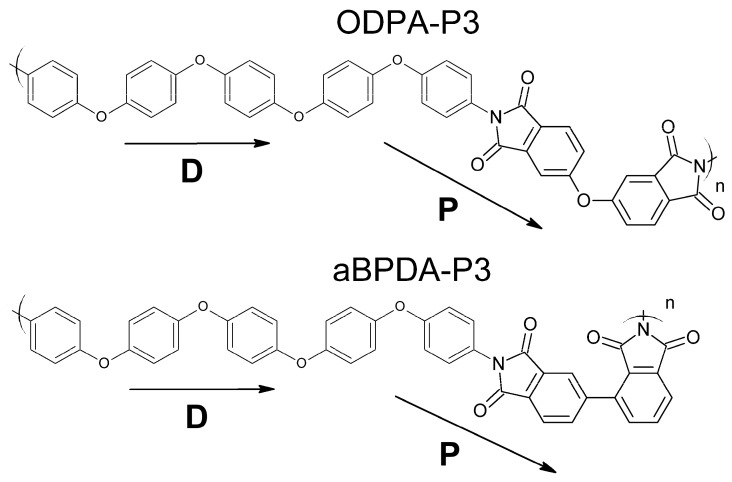
Chemical structure of the thermoplastic polyetherimide (PEI) repeat units: 3,3′,4,4′-oxydiphthalic dianhydride (ODPA)-P3 and 2,3′,3,4′-biphenyltetracarboxylic dianhydride (aBPDA)-P3. Arrows mark the vectors **D** and **P** aligned along phenylene and phthalimide planar moieties of the polyetherimide chains under study, for which the orientation to the nanotube axis was investigated in this paper. Phenylene rings in the moiety designated by the **D** vector can rotate out of a planar structure; however, such deviations from a planar structure are small, especially in the vicinity of the CNT surface, and phenylene rings are mostly coplanar.

To understand better how SWCNTs nucleate crystallization in amorphous thermoplastic polyetherimides, we studied SWCNT-filled nanocomposites based on ODPA-P3 and aBPDA-P3. The backbone of both polymers is almost identical with the exception of the dianhydrides used. 3,3′,4,4′-oxydiphthalic dianhydride (ODPA) induces a slight kink in the polymer backbone, whereas 2,3′,3,4′-biphenyltetracarboxylic dianhydride (aBPDA) induces a local 90° kink in the polymer backbone, which severely compromises the backbone linearity; see Figure 1.

In order to study the intermolecular structure at the polymer-SWCNT interface, we analyzed the orientation-related ordering of the PEI chain planar moieties shown by the vectors **P** or **D** in Figure 1. It should be noted that the phenylene rings designated by the vector **D** could form non-planar conformations due to the rotation of the rings. However, such deviations are rather small especially in the vicinity of the CNT surface, and both rings are mostly in a coplanar conformation.

Previously, we used atomistic molecular dynamics to explore the structural properties of nanocomposites based on amorphous R-BAPS containing polar sulfone groups (–SO_2_–) and partly crystallizable R-BAPB [50]. We showed that composites based on R-BAPB build up an ordered structure near the filler surface due to the orientation of the polymer chain planar fragments primarily along the nanoparticle surface [50,51,53]. At the same time, polyetherimide R-BAPS showed no orientation-related ordering in the corresponding composites. The obtained results correlate with the published experimental data on nanocomposites based on these PEIs [24,25]. However, the presence of a polar sulfone group in the repeat unit of the polyetherimide R-BAPS does not give a definitive answer to the question whether the lack of crystallization of this polyetherimide is related to the particular non-conformation behavior of the chains of this polymer or to specific dipole-dipole interactions between the polar groups. Therefore, in the present study, we simulated the structural properties of two PEIs, ODPA-P3 and aBPDA-P3, with a simpler molecular backbone composition, i.e., only phenyl rings, ether linkages and phthalimide units are present. The similarity of their chemical structure and the absence of the sulfone groups will enable us to provide modeling insights into the initial PEI SWCNT-induced crystallization.

The main goal of the present study is to elucidate the underlying molecular mechanism responsible for the initial stages of SWCNT–ODPA-P3 crystallization based on microsecond simulations. In studies of polymer structural properties, typical simulation times do not exceed 100 ns; however, this is not enough for heterocyclic polymers, which require much longer simulation times [55]. To validate the simulation approach and the chosen force field, we started with reproducing the known experimental differences between the structural properties of ODPA-P3 and aBPDA-P3 in the presence of SWCNTs. Then, with the help of excluded volume interaction parameters, we compared the influence of spatial restrictions, due to the nanotube presence and π–π stacking with the initial stage of SWCNT–ODPA-P3 crystallization.

## 2. Computer Simulations

We simulated two nanocomposites based on ODPA-P3 and aBPDA-P3 polyetherimides. Their repeat unit consist of two fragments, 3,3′,4,4′-oxydiphthalic dianhydride (ODPA), 2,3′,3,4′-biphenyltetracarboxylic dianhydride (aBPDA), and diamine 1,4-*bis*[4-(4-aminophenoxy)phenoxy]benzene (P3). Both PEIs are reinforced with single-walled carbon nanotubes (SWCNTs). The molecular structures are shown in Figure 1.

### Models and Simulation Method

The initial configurations of ODPA-P3 and aBPDA-P3 were created using an approach previously developed by us [50,51]. To start, 27 polymer chains with a polymerization degree of *N*_p_ = 8 were randomly placed into a periodic cell. After that, a SWCNT with a chirality of (5,5), a diameter of 0.7 nm, and a length of 4.7 nm comprised of 400 carbon atoms was placed into the center of the cell. In order to maintain the proper carbon atom valency, hydrogen atoms were attached at the carbon atoms positioned at both carbon nanotube termini. No partial charges were assigned to atoms in the CNT. The CNT model is similar to the one used in our previous studies [50,51].

The initially-generated nanocomposite was compressed for 26 ns. To relieve residual stresses after the compression step, an annealing process was performed, consisting of three cycles of temperature drop and increase, within the range of 800 to 300 K. After the annealing step, the systems were instantly cooled down from *Т* = 800 K to 600 K followed by a simulation for 2 μs at a temperature exceeding the glass transition point (*T*_g_) of the polyetherimides. This simulation timescale is close to the slowest relaxation processes related to moving a polymer coil over distances comparable to its own size, as was shown for the R-BAPB and R-BAPS aromatic polyetherimides with polymerization degree *N*_p_ = 8 at temperature 600 K [55,62]. During equilibration, the average sizes of polymer chains reached their equilibrium values, which are in good agreement with the theoretical estimates; see Figure S1 in the Supplementary Materials. To investigate the polymer structure at the polymer-CNT interface, the 1 μs-long production runs were performed at *T* = 600 K and *T* = 580 K. The choice of said temperatures was motivated by previous experimental results [63]. In fact, experimental results reported by Hegde et al. [63] showed that the melting point of the nanocomposite based on ODPA-P3 with the minimum SWCNT loading (≈0.1 vol %) is about 580 K. Therefore, the simulations were performed in the temperature range close to the experimental melting point.

The Gromacs package (Royal Institute of Technology and Uppsala University, Sweden; Version 4.6.7) [65,66] was used to perform MD using the force field Gromos53a5 [67]. This force field was successfully used earlier to simulate the structural, thermal, and mechanical properties of thermoplastic PEIs [50,51,52,53,54,55,56,57,58,59,60,61,62].

## 3. Results and Discussion

At the initial simulation stage, as in our previous study [50], a test was carried out to estimate the influence of the partial charges on the structural properties of thermoplastic PEIs. Assigning partial charges substantially increases the demand on computational resources [50,55,58]. Thus, in our study, we performed only 100 ns-long simulations with partial charges after a 1.5 µs-long equilibration procedure.

The values of partial charges were calculated using the Hartree–Fock quantum chemical method in the basic set of wave functions 6-31G* [58]. The electrostatic interactions during MD simulations were calculated using the Particle-mesh Ewald (PME) scheme [68] with parameters similar to our previous study [54].

As for the R-BAPB [50], the results obtained confirmed only a weak impact of the partial charges on the polymer layer structure at the interface; see Figure S3 in the Supplementary Materials. This may be attributed to the fact that the values of partial charges on the carbon nanotube atoms equal zero, and the explicit partial charges in the polymer have practically no influence on the general pattern of the structural ordering of the PEI planar fragments near the CNT surface. Therefore, it was decided to ignore the polymer partial charges, which allowed for a significant reduction in simulation time.

To investigate the PEI structure, the pair distribution functions were calculated for different groups of atoms

(1) $$g_{AB}\left(r\right)=\frac{1}{N_{A}\langle \rho_{B}\rangle }\sum_{i\in A}^{N_{A}}\sum_{j\in B}^{N_{B}}\frac{\delta \left(r_{ij}-r\right)}{4\pi r^{2}}$$

where $N_{A}$ and $N_{B}$ are numbers of type A and B atoms, respectively, $\langle \rho_{B}\rangle$ is the mean density of type B atoms located inside the sphere of radius r from the atom А, $r_{ij}$ is the distance between two atoms, and δ is the Dirac delta function.

The distant-dependent density distribution of the PEIs monomers has been calculated:

(2) $$\rho \left(r\right)=\frac{m\left(r\right)}{\pi h_{CNT}\times \Delta r\times \left(2r+\Delta r\right)}$$

where m(r) is the polymer mass in the cylinder layer Δr at a distance r from the CNT axis. The cylinder layer height is determined by the CNT length, $h_{CNT}$ ≈ 4.7 nm.

**Figure 2:**
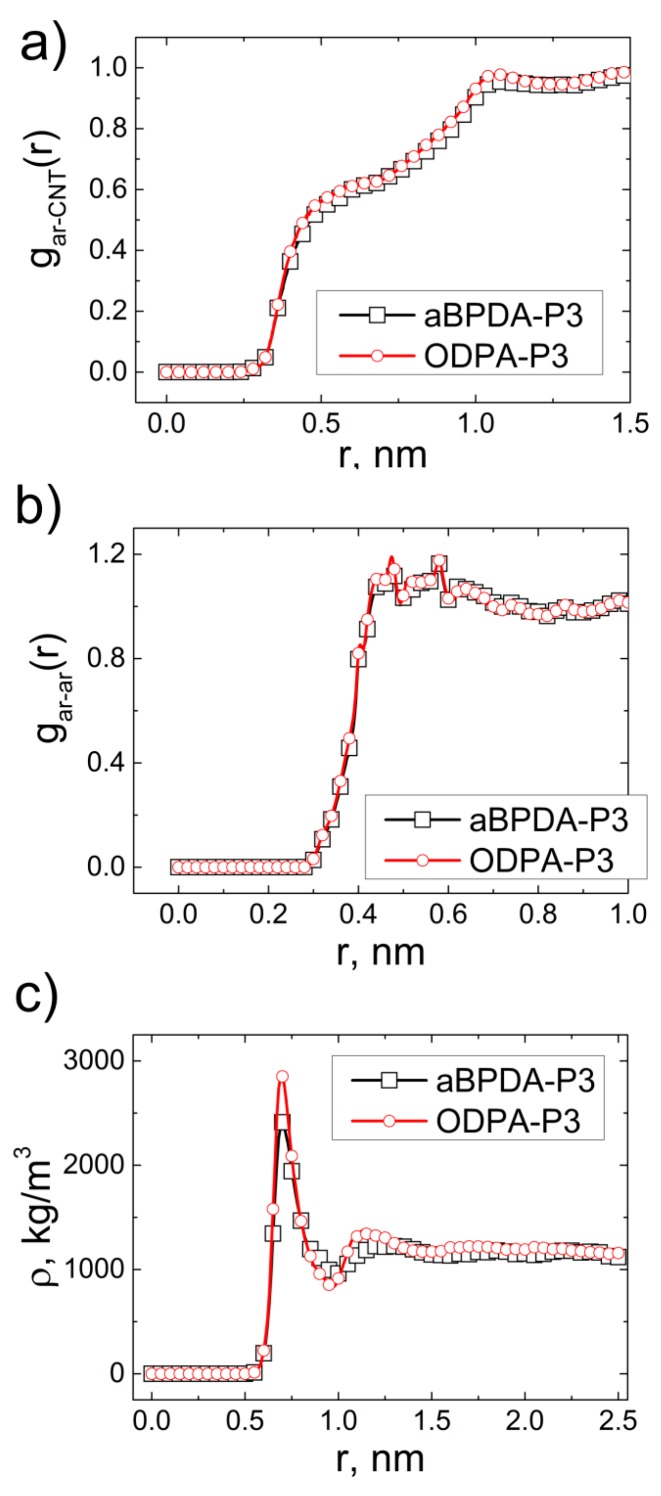
Pair distribution functions for the nanocomposites based on thermoplastic polyetherimides aBPDA-P3 and ODPA-P3 (**a**) between the carbon atoms of all aromatic rings and CNT atoms $g_{ar-CNT}\left(r\right)$ and (**b**) between the carbon atoms of the aromatic rings in relation to each other $g_{ar-ar}\left(r\right)$; (**c**) the dependence of the aBPDA-P3 and ODPA-P3 density on the distance from the CNT axis. *T* = 600 K in all cases.

The pair distribution functions between all carbon atoms in the polyetherimide aromatic rings and carbon nanotube $g_{ar-CNT}\left(r\right)$, all carbon atoms in the aromatic rings $g_{ar-ar}\left(r\right)$, as well as the dependence of the PEI density on the distance from the CNT axis ρ(r) have been calculated; see Figure 2.

The pair distribution functions $g_{ar-CNT}\left(r\right)$ and $g_{ar-ar}\left(r\right)$ of the two PEIs do not differ much from each other; see Figure 2a,b. The pair distribution functions $g_{ar-CNT}\left(r\right)$, as usual [50,69,70], demonstrate two shoulders responsible for interactions between the nanotube and the polymer chains [50]. The vertices of the curves for both polyetherimides are located at the same distance; see Figure 2a. The pair distribution functions $g_{ar-ar}\left(r\right)$ (Figure 2b) show a similar trend for both PEIs. Two local peaks on $g_{ar-ar}\left(r\right)$ at *r* ≈ 0.45 nm and *r* ≈ 0.58 nm may be related to interactions between the carbon atoms of the heterocyclic rings of the PEI and the CNT [57]. The dependences of ρ(r) near the CNT surface for the two PEIs are quite different; see Figure 2c. The density of the ODPA-P3 chains at a distance of the first peak (*r* = 0.75 nm) near the CNT surface is much higher than the density of the aBPDA-P3 chains. Besides, the dependence ρ(r) of ODPA-P3 has a more pronounced second maximum, which shows that the ODPA-P3 chains adsorb more strongly onto the CNT surface than the aBPDA-P3 chains.

In order to study the intermolecular structure at the polymer-SWCNT interface, we analyzed the orientation-related ordering of the phenylene and phthalimide planar moieties in the PEI chains, represented by the **D** and **P** vectors in Figure 1 correspondingly, and calculated the order parameters, S(r)

**Figure 3:**
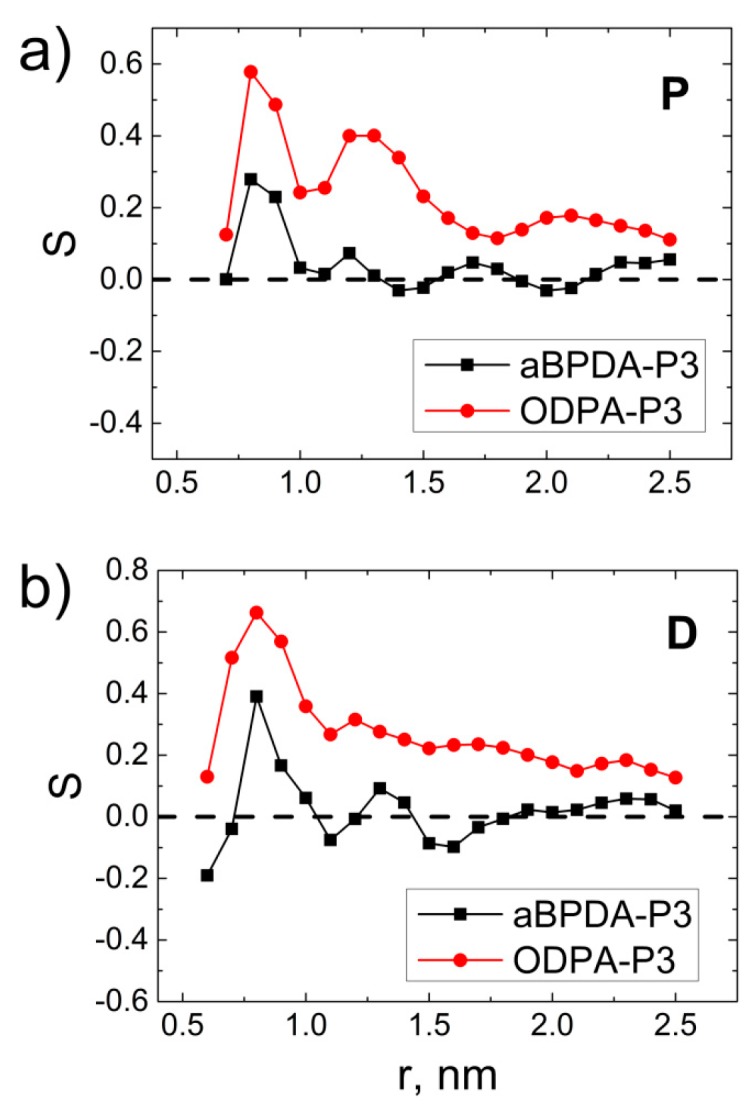
Order parameter for the vectors (**a**) **P** and (**b**) **D** for the nanocomposites based on ODPA-P3 and aBPDA-P3 at *Т* = 600 K as a function of the distance from the nanotube axis after a 1-μs production run.

(3) S(r)=32〈cos2θ(r)〉−12

where *r* is the distance from the nanotube axis to the polymer chain planar moiety and θ is the angle between the vector **P** or **D** directed along the planar moiety of the PEI chains and the carbon nanotube axis, Figure 3.

The order parameters S(r) calculated for the **P** and **D** vectors showed that at *T* = 600 K, the planar fragments of ODPA-P3 orient themselves more strongly along the CNT surface. The first peak in the S(r) dependence for both polyetherimides is attributed to the orientation of the PEI planar fragments directly at the polymer–SWCNT interface. The order parameter for the **P** vector in ODPA-P3 shows the second peak at a distance of about 1.5 nm, which is absent in the S(r) dependence of aBPDA-P3. This fact demonstrates a rather strong structural ordering of the ODPA-P3 planar fragments even far from the nanotube surface. As opposed to the S(r) for ODPA-P3, the dependence S(r) for aBPDA-P3 has one maximum only and rapidly decreases to zero at increasing distances from the nanotube surface.

The emerging structural ordering of the ODPA-P3 polymer chain fragments may be interpreted as the initial crystallization stage of this polyetherimide [71,72]. In fact, previous results allowed the interpretation of polymer crystallization through several stages. First of all, polymer chain fragments orient themselves along the nanofiller surface. Further on, polymer chains orient and order themselves on larger spatial scales, which are accompanied by the order parameter growth in the system. This is followed by the entire ordering of polymer chains in the system, accompanied by an increase in the system local density and the formation of a structure similar to a crystalline one. Therefore, the structural ordering of the ODPA-P3 planar fragments near the CNT surface may be regarded as the initial polymer crystallization stage on local spatial scales. Similar results were obtained in our previous study for PEI R-BAPB [50].

**Figure 4:**
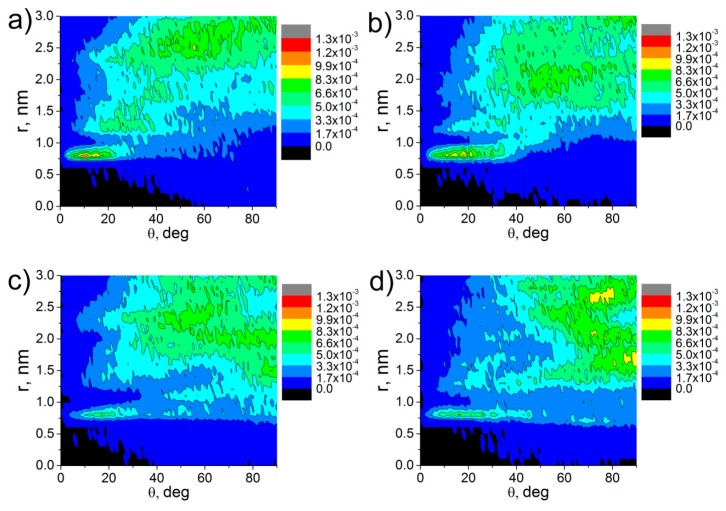
Diagrams of the distribution of the θ angle between the CNT axis and vectors **P** (**a**,**c**) and **D** (**b**,**d**) of thermoplastic PEIs: ODPA-P3 (**a**,**b**) and aBPDA-P3 (**c**,**d**) at *Т* = 600 K after a 1-μs production run.

For the systems under the study, we also analyzed the distributions of the orientation angles θ of the vectors **P** and **D** relative to the CNT axis; the results are shown in Figure 4.

The orientation angle distribution (Figure 4) demonstrates that in composites based on ODPA-P3, several layers in the vicinity of the CNT surface are forming where the PEI chain planar moieties are aligned along the CNT axis. Furthermore, the maximum in the distribution diagram is reached at the values of the angles θ within five to 20 degrees, and the structural ordering of the ODPA-P3 planar moieties extends to a distance of about 3 nm from the CNT axis (Figure 4a,b), i.e., considering the periodic boundary conditions, the orientation of the planar moieties takes place almost across the entire volume of the simulation cell. At the same time, the diagrams of the angle distribution between the carbon nanotube axis and vectors **P** and **D** for aBPDA-P3 do not confirm the orientation of planar fragments near the CNT surface at a simulation time of ~1 µs. From Figure 4c,d, it is clear that the angles θ between the vectors **P** and **D** and the CNT axis are distributed practically homogeneously even near the CNT surface.

**Figure 5:**
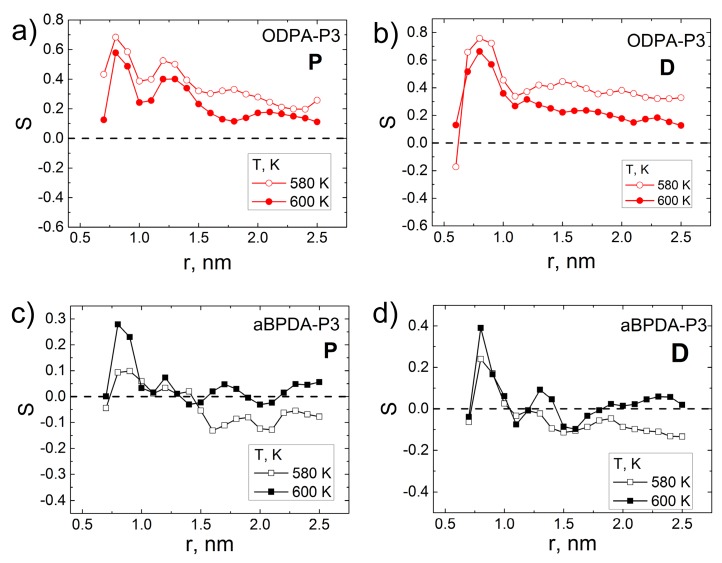
Order parameter S(r) for the vectors **P** (**a**,**c**) and **D** (**b**,**d**) of ODPA-P3 (**a**,**b**) and aBPDA-P3 (**c**,**d**) and at temperatures of 580 K and 600 K subject to the distance from the CNT axis after a 1-µs production run.

The absence of structural ordering of the aBPDA-P3 planar moieties near the CNT surface may be caused by the high temperatures used. Therefore, we studied the nanocomposite intermolecular structure at a lower temperature of *Т* = 580 K. To the end of the equilibration run at *Т* = 600 K, the temperature was instantly reduced to 580 K, and additional simulations of the system have been carried out for 1 µs. After that, the order parameter S(r) was calculated for the vectors **P** and **D** again; see Figure 5.

The results obtained demonstrate that in the case of aBPDA-P3, the temperature drop from 600 to 580 K does not lead to any structural ordering near the CNT surface; see Figure 5. However, the temperature drop impacts the molecular structure of the ODPA-P3-based composite significantly. For this polymer, the temperature decrease leads to the increase in the second maximum value in the S(r) dependence and higher values of S(r) at larger distances r from the CNT surface.

**Figure 6:**
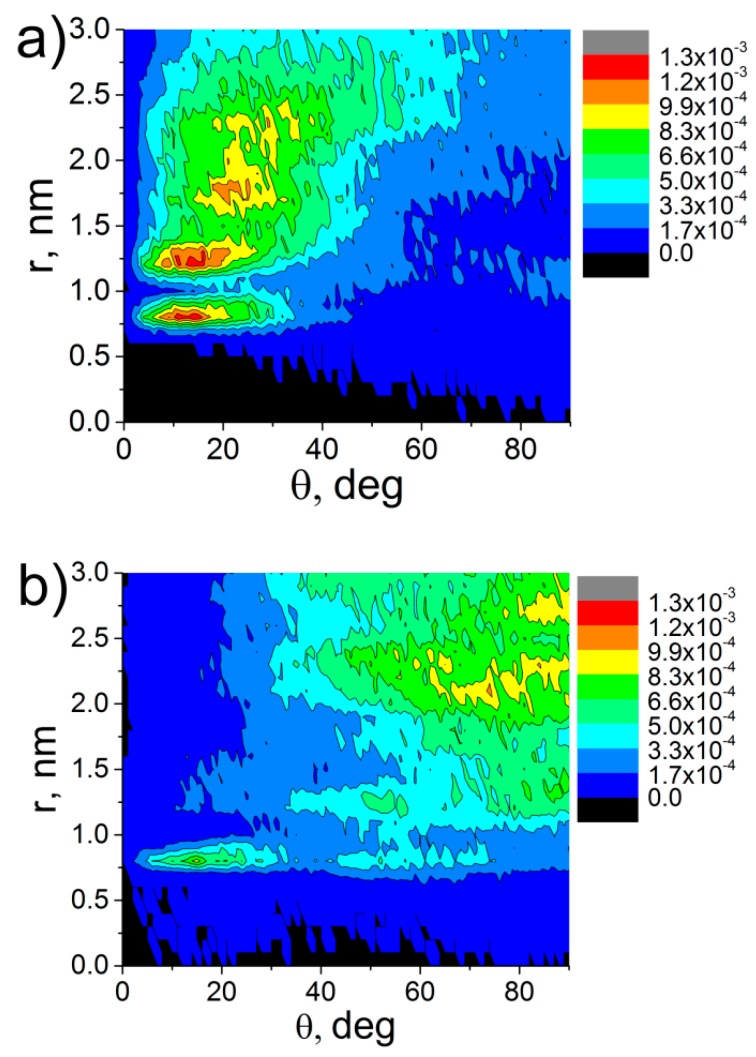
Distribution diagrams of the angles between the nanotube axis and vector **P** of the thermoplastic PEIs: (**a**) ODPA-P3 and (**b**) aBPDA-P3 at *T* = 580 K after a 1-μs production run.

At a temperature of 580 K, we also calculated the distributions of the orientation angles *θ* between the planar moieties of the repeat unit of ODPA-P3 and aBPDA-P3 after a 1-μs production run; see Figure 6.

The analysis of the results obtained shows that the maximum distributions of orientation angle for ODPA-P3 composites shift to lower θ values after the temperature drop (compare Figure 6a and Figure 4a). Furthermore, the formation of more pronounced second layer at a distance of ≈1.25–1.5 nm from the nanotube axis with planar moieties oriented largely at an angle of 25 degrees is observed, as well as the formation of oriented layers at larger distances. At the same time, the analysis of the distributions of orientation angles θ between the aBPDA-P3 planar fragments and the CNT axis at a temperature of 580 K failed to confirm the structural ordering of this polymer near the CNT surface; see Figure 6b.

**Figure 7:**
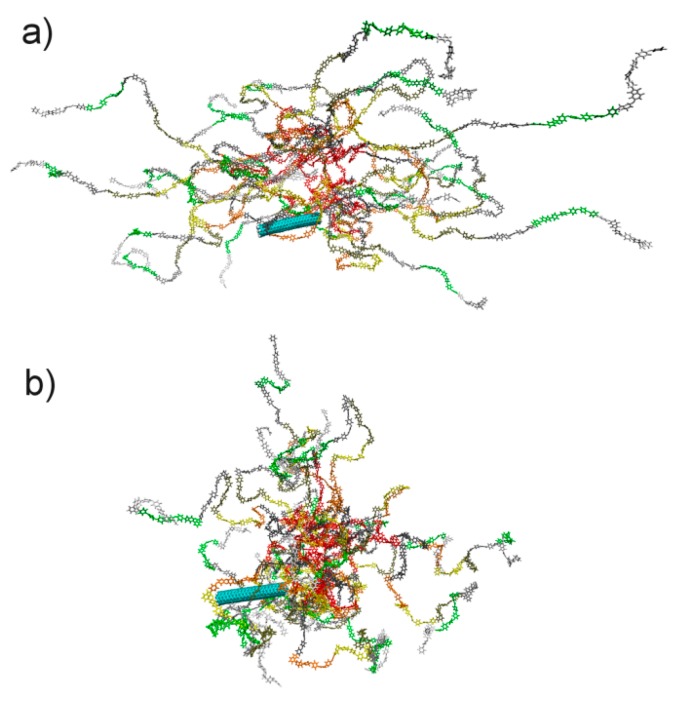
Instant configurations of SWCNT-PEI composites based on thermoplastic polyetherimides (**a**) ODPA-P3 and (**b**) aBDPA-P3 after a 1-μs simulation at a temperature of 580 K.

Notably, different structural properties of nanocomposites based on thermoplastic PEIs ODPA-P3 and aBPDA-P3 may also be observed during the analysis of their instant configurations. Figure 7 shows snapshots of the PEIs under study after a 1-μs simulation at a temperature of 580 K.

The analysis of Figure 7a suggests that chains of crystallizable polyetherimide ODPA-P3 orient themselves near the surface of the CNT alongside the nanofiller surface. At the same time, there is no orientation of planar moieties in the amorphous polyetherimide aBPDA-P3 near the CNT surface; see Figure 7b.

**Figure 8:**
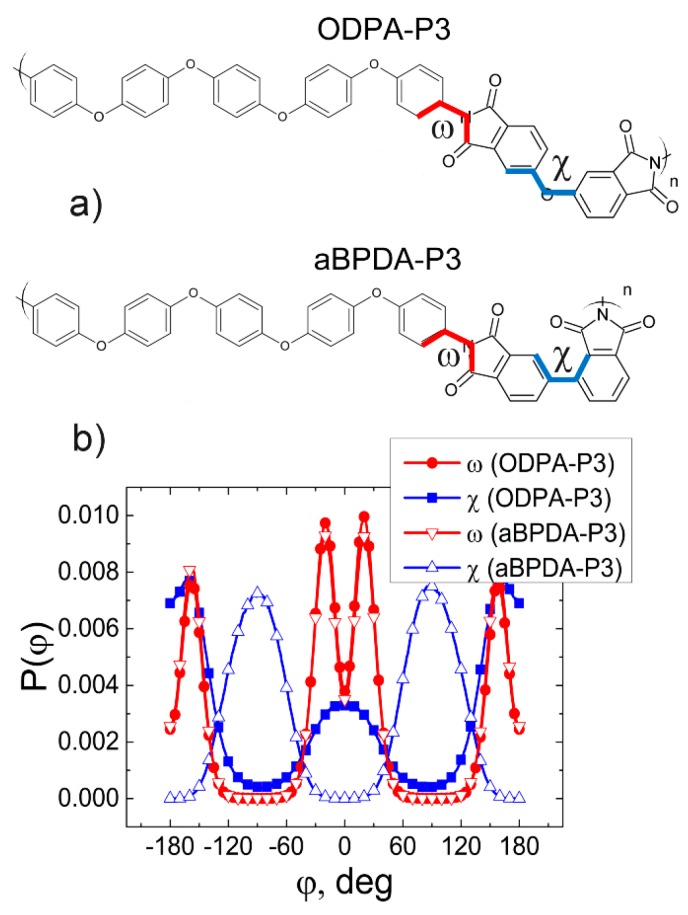
(**a**) Definition of dihedral angles *ω* and *χ* in the investigated ODPA-P3 and aBPDA-P3 polyetherimides. Red and blue lines mark the bonds for which the dihedral angles were calculated. (**b**) Distributions P(φ) of the dihedral angles *ω* and *χ* of ODPA-P3 and aBPDA-P3 at the temperature of 580 K.

As opposed to aBPDA-P3, structural ordering of the ODPA-P3 planar moieties is due to the different chemical structures of their dianhydride moieties. In this study, we calculated the distributions P(φ) of the dihedral angles of internal rotation (*ω*, *χ*) in ODPA-P3 and aBPDA-P3; see Figure 8a.

The analysis of the results obtained shows that distributions of dihedral angles *ω* of ODPA-P3 and aBPDA-P3 almost fully coincide. The most probable values of the angles *ω* in both polyetherimides amount to approximately 10° and 160°. However, distributions of dihedral angles *χ* of the two polyetherimides differ from each other. For the aBPDA-P3, the most probable value of the dihedral angle *χ* is equal to 90°, and the most probable value of this angle for the ODPA-P3 amounts to approximately 160°; see Figure 8b. Therefore, the phthalimide moieties in aBPDA are unfolded perpendicularly in relation to each other, which prevents the structural ordering of the planar moieties near the CNT surface. To this end, this spatial confinement both influences the adsorption of the polymer chain to the CNT surface in the dianhydride fragment and prevents the structural ordering of phenylene rings of the diamine fragment of aBPDA-P3. At the same time, the dianhydride fragment of ODPA-P3 has practically a planar structure facilitating the orientation of phthalimide moieties and phenylene rings along the CNT surface.

Therefore, we succeeded in simulating the experimental effect reported previously on the ODPA-P3 crystallization initiation after the CNT incorporation [63,64]. Thus, results obtained here provide at least the correct choice of the force field to predict differences in the PEIs’ structural properties.

As mentioned in the Introduction, the ordering of planar moieties of the polymer repeat unit may be regarded as the initial crystallization stage of the thermoplastic polyetherimide ODPA-P3 [71,72]. It is assumed that the molecular mechanism of the initial crystallization stage of the ODPA-P3 is determined by either the CNT presence or influence of π–π interactions between CNT and ODPA-P3 atoms. Then, we will try to answer the question as to what the main factor is in the ordering process of ODPA-P3 planar moieties towards the CNT surface.

Notably, the correct consideration of π–π interactions in molecular dynamics is a non-trivial task when classical mechanical force fields are used, which do not account for quantum effects. Nevertheless, since in reality the molecular dynamics force fields are semi-empirical, π–π interactions are efficiently accounted for by excluded volume interactions. Moreover, it was shown that simulations with classical force fields allow reproducing structural properties of molecular systems determined by π–π interactions [73]. In our previous studies [50,51,58], we have investigated the influence of electrostatic interactions on the structure of nanocomposites based on the polyetherimide R-BAPB. Ordered structures in these systems were formed during the simulation both with and without partial charges. Since, as it was shown in these studies, the ordering is related to the orientation of aromatic fragments of the polyetherimide chains towards the CNT axis and there are π–π interactions between them in real systems, it may be acceptable to state that π–π are quite accurately accounted for in the Gromos53a5 force field through excluded volume interactions between specific atom types.

**Figure 9:**
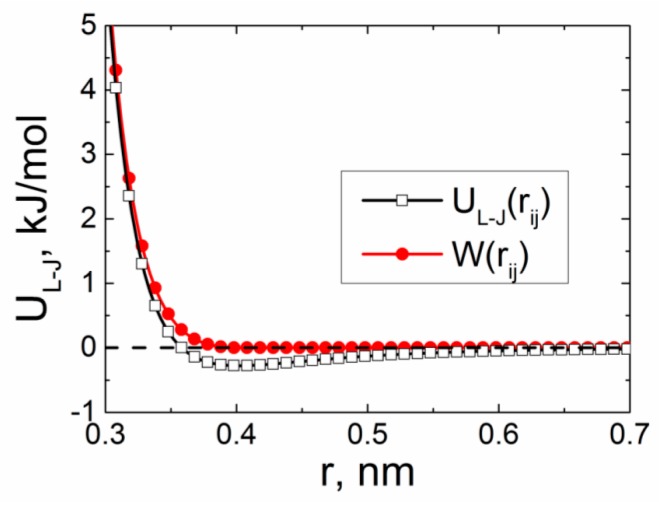
Lennard–Jones $U_{L-J}\left(r_{ij}\right)$ and Weeks–Chandler–Anderson $W\left(r_{ij}\right)$ potentials between the carbon atoms of the CNT and ODPA-P3 using the initial values of the parameters *ε* and *σ* of the van der Waals energy from the Gromos53a5 force field. The dashed line in the figure marks the zero value of the van der Waals energy.

Thus, changes in the form and parameters of the excluded volume interactions between the CNT atoms and polymer chain atoms in the simulation allow for investigating the influence of each of the two effects in question on the polymer-CNT interface structure. Therefore, we studied how changes in the form and parameters of the excluded volume interactions between the carbon atoms of the polymer and the CNT influence the structural ordering of the ODPA-P3 planar fragments near the CNT surface. In the force field Gromos53a5 [67,74], the excluded volume interactions are set using the Lennard–Jones (LJ) potential:

(4) $$U_{L-J}\left(r_{ij}\right)=4\varepsilon \left({\left(\frac{\sigma }{r_{ij}}\right)}^{12}-{\left(\frac{\sigma }{r_{ij}}\right)}^{6}\right)$$

where *ε* is the potential well depth, *σ* is the distance at which $U_{L-J}\left(r_{ij}\right)$ becomes equal to zero (van der Waals radius), and $r_{ij}$ is the distance between the *i*-th and *j*-th atoms. At the beginning, to verify the impact of the geometric confinement through the CNT in the nanocomposites, during the initial crystallization stage of thermoplastic polyetherimides, we performed a simulation of composites based on ODPA-P3 using the modified LJ potential written in the form of the pure repulsive Weeks–Chandler–Anderson (WCA) potential, $W\left(r_{ij}\right)$ [75]:

(5) $$W\left(r_{ij}\right)=\left\{ \begin{array}{ll} U_{L-J}\left(r_{ij}\right)+\varepsilon ,\; & r\leq r_{0}\\ 0,\; & r>r_{0} \end{array}\right.$$

where $r_{0}$ = $\sqrt[{6}]{2}\sigma$ is the distance corresponding to the minimum of the function $U_{L-J}\left(r_{ij}\right)$. LJ and WCA potentials are given in Figure 9.

The use of the WCA potential to describe interactions between the carbon atoms of the CNT and heterocyclic rings of ODPA-P3 enables us to exclude the attraction of planar fragments to the CNT surface due to π–π interactions. In this case, the orientation of the ODPA-P3 planar moieties could be the result of spatial confinements promoted by the CNT presence.

**Figure 10:**
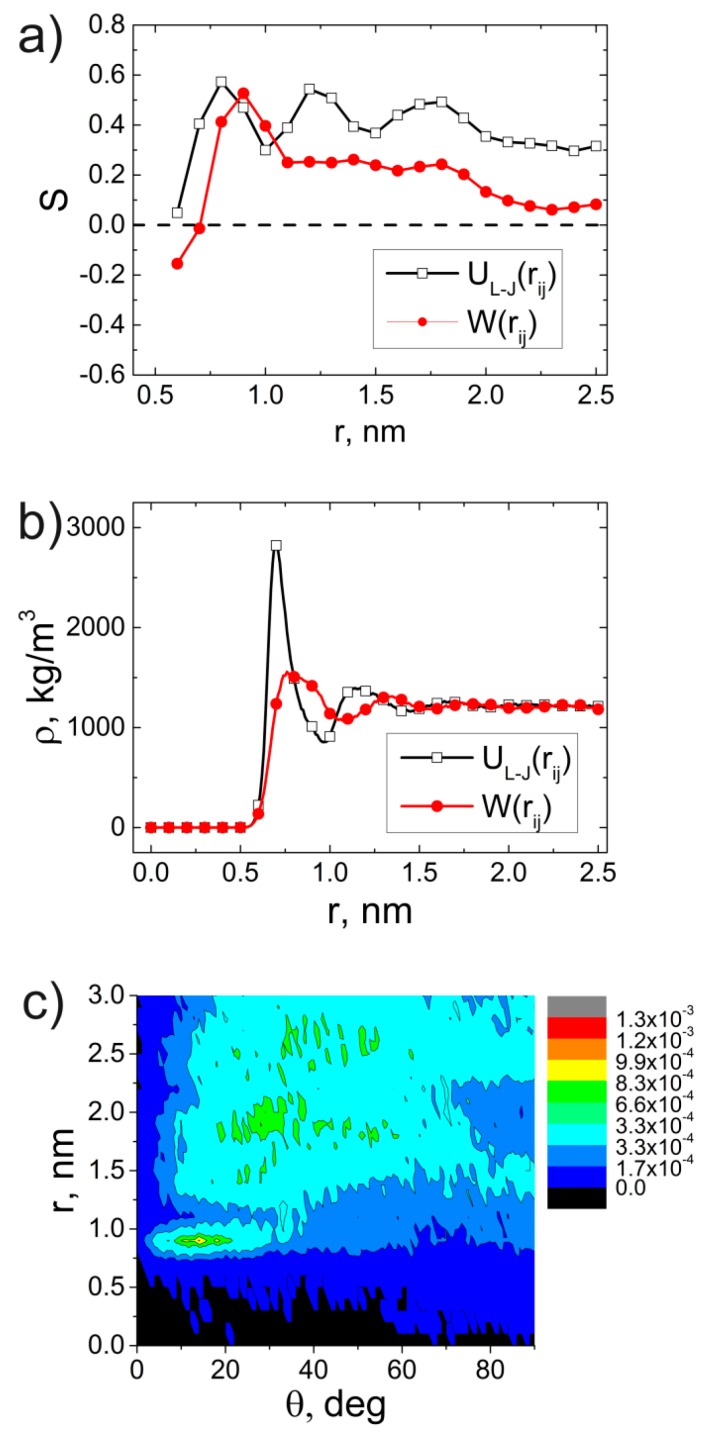
Dependences of the order parameter of the vector **P** (**a**) and density (**b**) of the given nanocomposites based on ODPA-P3 for the different description of the van der Waals energy in the form of the Lennard–Jones $U_{L-J}\left(r_{ij}\right)$ and Weeks–Chandler–Anderson $W\left(r_{ij}\right)$ potentials; (**с**) distribution of the orientation angles θ between the vector **P** and CNT surface for the Weeks–Chandler–Anderson potential at *T* = 580 K after the 1-µs simulation.

The change of the form of excluded volume interaction potential was followed by additional simulation for 1 µs. Then, we calculated the dependences of the order parameter S(r) and density ρ(r) of the ODPA-P3 on the distance from the CNT axis and distributions of the orientation angles θ between the ODPA-P3 planar moieties and CNT axis; see Figure 10.

The analysis of the data presented in Figure 10 allows us to conclude that 1-µs simulation using the WCA potential instead of the LJ potential leads to the destruction of the structural ordering of the ODPA-P3 planar segments near the CNT surface. The dependence of the order parameter S(r) on the distance features a drop of the second and third maximum values and the shift of their position for a larger distance away from the CNT axis. Similar conclusions can be formulated from the analysis of the dependence ρ(r) of ODPA-P3. The dependence ρ(r) obtained using the WCA potential shows the position of the first and the second maxima significantly lower and shifted to larger r values relative to the position of the maxima in the dependence ρ(r) calculated using the LJ potential, which provides evidence that the ODPA-P3 polymer chains cease to be attracted to the CNT surface.

Comparative analysis of the distributions of the orientation angles θ of the ODPA-P3 planar moieties near the CNT surface for the WCA potential (Figure 10с) and LJ potential (Figure 6a) shows a practically complete disappearance of the structural ordering of the ODPA-P3 planar fragments in the case of the WCA potential. The analysis of the dependence S(r) and distributions of the orientation angles *θ* of the ODPA-P3 planar fragments relative to the CNT also confirms the disappearance of all structural ordering of the ODPA-P3 planar moieties near the CNT surface; see Figures S4 and S5 in the Supplementary Materials. Notably, when the simulation was extended to 2 µs, there was still no structural ordering of the ODPA-P3 planar moieties near the CNT surface; see Figure S5 in the Supplementary Materials. This confirms the lack of interaction between the carbon atoms of the PEI and CNT, i.e., insufficient strength of π–π interactions at the polymer-CNT phase interface. In the simulation, this leads to the disappearance of the structural ordering of the polymer planar moieties near the CNT surface. Thus, spatial confinements are not the determining factor for initiation of ODPA-P3 crystallization.

For an additional check of the influence of π–π interactions on the polymer-CNT interphase structure, we performed simulations with varying depth of the potential well ε of the interaction energy (4) between the carbon atoms of the CNT and the ODPA-P3 heterocyclic rings. As was shown in the present study, the orientation of planar moieties near the CNT surface has been found in computer simulation both with and without taking into account electrostatic interactions. Since, the ordering is related to the orientation of aromatic fragments of the PEI chains towards the CNT axis and there are π–π interactions between them in real systems, it may be acceptable to state that π–π interactions are quite accurately accounted for in the Gromos53a5 force field through volume interactions between specific atom types in agreement with results of our previous investigations of the structural properties of R-BAPB-based nanocomposites [50,51,58].

**Figure 11:**
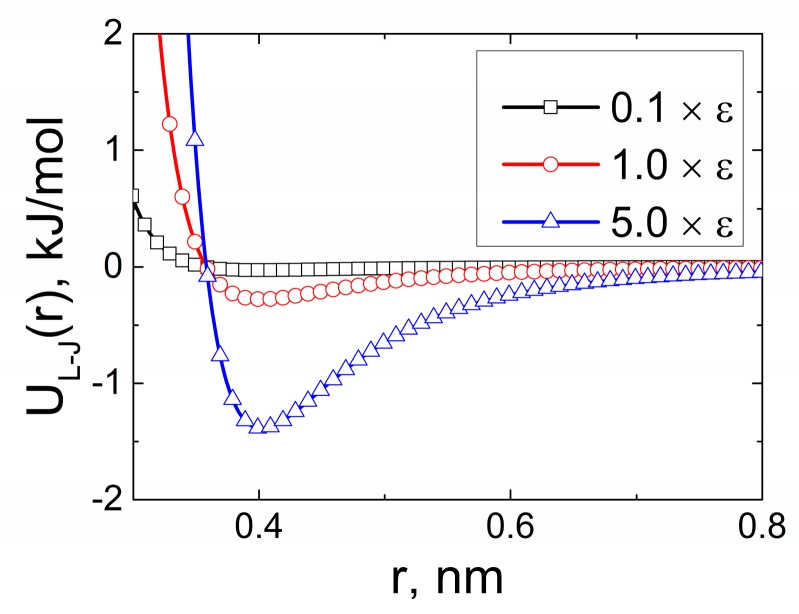
Van der Waals energy $U_{L-J}\left(r_{ij}\right)$ for the varying potential well depth *ε* multiplied by 0.1- and five-times as compared to the initial parameters of the Gromos53a5 force field (ε=1 ).

Varying the value of the parameter *ε*, we sought to efficiently relax or enhance the π–π interactions at the polymer-CNT phase interface. Figure 11 shows dependences $U_{L-J}\left(r_{ij}\right)$ for various values of *ε* used in our study. At that, the van der Waals radius value *σ* of the carbon atoms remained constant.

**Figure 12:**
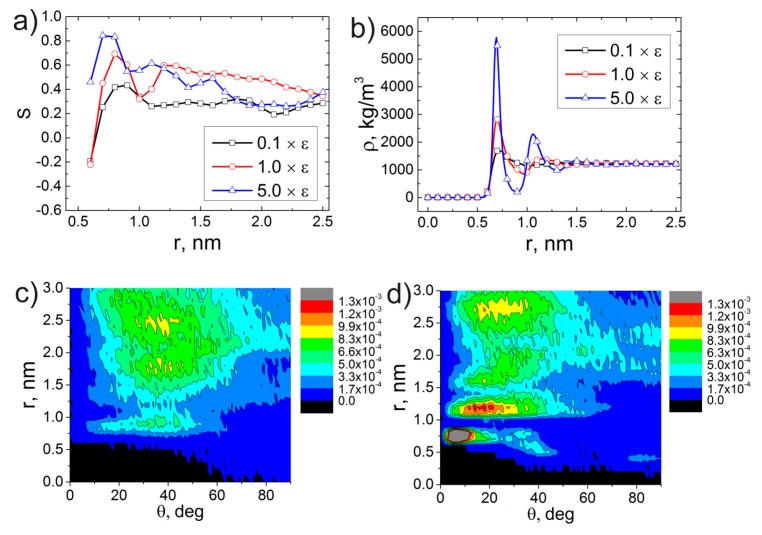
Dependences of the (**а**) order parameter S(r) for the vector **P** and (**b**) density ρ(r) on the distance from the CNT axis for varying values of the parameter ε. Distributions of angles θ between the vector **P** and CNT axis at the increase in the potential well depth ε by (**с**) 0.1- and (**d**) five-times as compared to the initial parameters of the Gromos53a5 force field after a 1-µs production run.

Changes in the potential well depth *ε* of the excluded volume interactions between the carbon atoms of the CNT and ODPA-P3 were followed by the additional simulation for 1 µs. Further, we investigated the structural properties of ODPA-P3. To this end, we calculated S(r), ρ(r) and distributions of the orientation angles θ between the PEI planar fragments and the CNT axis; see Figure 12.

The analysis of the dependence of the order parameter S(r) for the vector **P** shows that the 10-times reduction of *ε* leads to ODPA-P3 planar fragments moving away from the CNT surface. The dependence S(r) has only one maximum, which slowly decreases when the distance is growing; see Figure 12a. This may be attributed to the fact that after a 10-times reduction of the potential well depth ε, the ODPA-P3 atoms cannot adsorb on the CNT surface due to thermal fluctuations. When the parameter ε is increased by five-times, the dependence S(r) demonstrates the rise of the first maximum height, which confirms the fact that the ODPA-P3 heterocyclic rings find it more energetically favorable to orient themselves near the CNT surface. The analysis of the dependence ρ(r) for varying *ε* values shows that the increase in *ε* also causes the height of the first and second maxima to grow in the dependence ρ(r) of ODPA-P3, as well; see Figure 12b. With *ε* increasing, this polyetherimide starts to be more strongly attracted to the CNT surface. The increase or reduction of the potential well depth *ε* between the carbon atoms of the CNT and ODPA-P3 influences both the structural ordering of the ODPA-P3 planar moieties near the CNT surface and the polymer density near the CNT, i.e., on the adsorption of polymer chains on the CNT surface. These conclusions are substantiated also by the analysis of the distribution of orientation angles θ of the PEI planar fragments relative to the CNT axis. The results obtained have demonstrated that the reduction of the parameter *ε* by 10-times in the distribution of the orientation angles θ (Figure 12с) causes the second and third ordered polymer layers near the CNT surface to disappear, whereas these layers were observed earlier using the standard parameters of the force field; see Figure 6a. In the case of the five-times increase in the parameter ε, distributions of the orientation angles θ feature the first peak at a distance of ≈0.75–0.8 nm, where the planar fragments of the ODPA-P3 repeat unit are oriented at an angle of ~10°, and the second peak at a distance of ≈1.25–1.5 nm, where the planar moieties are oriented at an angle of ≈20°; see Figure 12d. The analysis of the results obtained shows a strong influence of specific π–π interactions of the polymer-CNT interface structure. Similar conclusions are suggested by the analysis of the dependences S(r) and distribution angles *θ* between the vector **D** and the CNT axis; see Figure S6 in the Supplementary Materials. Notably, if a simulation accounts for the attraction between the carbon atoms of the CNT and polymer, even in the case of the 10-times reduction of the parameter S(r) as compared to its initial value in the force field, weak structural ordering of the ODPA-P3 planar fragments still remains near the CNT surface after 1 µs.

Therefore, structural ordering of the ODPA-P3 planar fragments near the CNT surface is the result of π–π interactions between the carbon atoms of the CNT aromatic rings and polymer heterocyclic rings. Regarding this structural ordering as the initial stage of the thermoplastic ODPA-P3 crystallization due to the nucleating action of the carbon nanofiller, it can be affirmed that the presence of π–π interactions between the heterocyclic polymer and nanofiller surface initiates ODPA-P3 crystallization. We believe that this mechanism may be valid for other heterocyclic polymers, as well. Notably, these conclusions on the influence of π–π interactions on the initial stage of the thermoplastic polyetherimide ODPA-P3 crystallization correlate very well with the experimental findings on the adsorption of low molecular compounds with (locally) conjugated aromatic cycles on the CNT surface [76,77].

## 4. Conclusions

In the present study, we used microsecond-long atomistic molecular-dynamics simulations to investigate the molecular mechanisms responsible for the initial stage of thermoplastic polyetherimide crystallization. To this end, we simulated the structural properties of two amorphous thermoplastic polyetherimides, ODPA-P3 and aBPDA-P3, in the presence of SWCNTs. We observed that the planar phthalimide and phenyl rings of ODPA-P3 show an increase in order when they approach the CNT surface. This, in our opinion, can be considered as the initial stage of ODPA-P3 crystallization [63]. At the same time, no orientation ordering of the aBPDA-P3 planar fragments could be observed. The difference in structural properties of the PEIs investigated is mainly dictated by the difference in chemical composition of the dianhydride moiety.

To provide computational insights into the molecular mechanisms of the initial stage of PEI crystallization, we simulated the interface structure of the ODPA-P3 nanocomposite reinforced with CNTs. We explored two possible scenarios that could lead to ordering: the polymer orients itself near the CNT surface due to the spatial confinements or because of the strong influence of the π–π interactions at the polymer-CNT interface. It has been established here that in the absence of the attraction between the CNT surface and the PEI matrix, no structural ordering of the ODPA-P3 planar fragments near the CNT surface is observed. We have shown that by varying the strength of the van der Waals interactions between the CNT atoms and the polymer segments, it is possible to weaken or enhance the structural ordering of the ODPA-P3 planar fragments near the CNT surface. Therefore, we propose that the molecular mechanism of the thermoplastic PEI crystallization initiation, as a result of CNT incorporation, is the result of favorable π–π interactions between the heterocyclic polymer rings and the CNT surface where all carbon atoms are in sp^2^ hybridization state.

In the work presented in this paper, we did not consider CNT agglomeration effects, as this would have complicated our initial modeling efforts. CNT agglomeration (bundling) will most definitely affect polymer-CNT interactions and complicate PEI crystallization. We have already started to look at PEI crystallization in the presence of different CNT packing motives, e.g., crystallization at intersecting CNTs, and this work will be the basis for our next publication.

## Supplementary Materials

Supplementary Materials are available online at www.mdpi.com/2073-4360/9/10/548/s1.

## References

[1] Ishida H., Huang M.T. (1994). Molecular level study of the crystallization of a thermoplastic polyimide by infrared spectroscopy. J. Polym. Sci. B.

[2] Tamai S., Kuroki T., Shibuya A., Yamaguchi A. (2001). Synthesis and characterization of thermally stable semicrystalline polyimide based on 3,4′-oxydianiline and 3,3′,4,4′-biphenyltetracarboxylic dianhydride. Polymer.

[3] Ratta V., Ayambem A., McGrath J.E., Wilkes G.L. (2001). Crystallization and multiple melting behavior of a new semicrystalline polyimide based on 1,3-bis(4-aminophenoxy)benzene (TPER) and 3,3′,4,4′-biphenonetetracarboxylic dianhydride (BTDA). Polymer.

[4] Heberer D.P., Cheng S.Z.D., Barley J.S., Lien S.H.-S., Bryant R.G., Harris F.W. (1991). Crystallization and morphology of semicrystalline polyimides. Macromolecules.

[5] Fu Q., Livengood B.P., Shen C.C., Lin F., Harris F.W., Cheng S.Z.D., Hsiao B.S., Yeh F. (1998). Crystallization and phase behavior in nylon 6/aromatic polyimide triblock copolymers. Macromol. Chem. Phys..

[6] Srinivas S., Caputo F.E., Graham M., Gardner S., Davis R.M., McGrath J.E., Wilkes G.L. (1997). Semicrystalline polyimides based on controlled molecular weight phthalimide end-capped 1,3-bis(4-aminophenoxy)benzene and 3,3′,4,4′-biphenyltetracarboxylic dianhydride: Synthesis, crystallization, melting, and thermal stability. Macromolecules.

[7] Takekoshi T., Overberger C.G. (1990). Polyimides. Advances in Polymer Science.

[8] Cheng S.Z.D., Mittleman M.L., Janimak J.J., Shen D., Chalmers T.M., Lien H.S., Tso C.C., Gabori P.A., Harris F.W. (1992). Crystal structure, crystallization kinetics and morphology of a new polyimide. Polym. Int..

[9] Muellerleile J.T., Risch B.G., Rodrigues D.E., Wilkes G.L., Jones D.M. (1993). Crystallization behaviour and morphological features of LARC-CPI. Polymer.

[10] Moniruzzaman M., Winey K.I. (2006). Nanocomposites Containing Carbon Nanotubes. Macromolecules.

[11] Miltner H.E., Grossiord N., Lu K., Loos J., Koning C.E., Van Mele B. (2008). Isotactic polypropylene/carbon nanotube composites prepared by latex technology. Thermal analysis of carbon nanotube-induced nucleation. Macromolecules.

[12] Sandler J., Broza G., Nolte M., Schulte K., Lam Y.-M., Shaffer M.S.P. (2003). Crystallization of carbon nanotube and nanofiber polypropylene composites. J. Macromol. Sci. B.

[13] Assouline E., Lustiger A., Barber A.H., Cooper C.A., Klein E., Wachtel E., Wagner H.D. (2003). Nucleation ability of multiwall carbon nanotubes in polypropylene composites. J. Polym. Sci. B.

[14] Manchado M.A.L., Valentini L., Biagiotti J., Kenny J.M. (2005). Thermal and mechanical properties of single-walled carbon nanotubes-polypropylene composites prepared by melt processing. Carbon.

[15] Li L., Yang Y., Yang G., Chen X., Hsiao B.S., Chu B., Spanier J.E., Li C.Y. (2006). Patterning polyethylene oligomers on carbon nanotubes using physical vapor deposition. Nano Lett..

[16] Li L., Li C.Y., Ni C. (2006). Polymer crystallization-driven, periodic patterning on carbon nanotubes. J. Am. Chem. Soc..

[17] Xu G., Zhuang Y., Xia R., Cheng J., Zhang Y. (2012). Carbon nanotubes induced nonisothermal crystallization of ultrahigh molecular weight polyethylene with reduced chain entanglements. Mater. Lett..

[18] Chae H.G., Minus M.L., Kumar S. (2006). Oriented and exfoliated single wall carbon nanotubes in polyacrylonitrile. Polymer.

[19] Lu K., Grossiord N., Koning C.E., Miltner H.E., van Mele B., Loos J. (2008). Carbon nanotube/isotactic polypropylene composites prepared by latex technology: Morphology analysis of CNT-induced nucleation. Macromolecules.

[20] Phang I.Y., Ma J., Shen L., Liu T., De Zhang W. (2006). Crystallization and melting behavior of multi-walled carbon nanotube-reinforced Nylon-6 Composites. Polym. Int..

[21] Chatterjee S., Nüesch F.A., Chu B.T.T. (2011). Comparing carbon nanotubes and graphene nanoplatelets as reinforcements in polyamide 12 composites. Nanotechnology.

[22] Sandler J.K.W., Pegel S., Cadek M., Gojny F., Van Es M., Lohmar J., Blau W.J., Schulte K., Windle A.H., Shafferf M.S.P. (2004). A comparative study of melt spun polyamide-12 fibres reinforced with carbon nanotubes and nanofibres. Polymer.

[23] Yudin V.E., Svetlichnyi V.M., Shumakov A.N., Schechter R., Harel H., Marom G. (2008). Morphology and mechanical properties of carbon fiber reinforced composites based on semicrystalline polyimides modified by carbon nanofibers. Compos. A.

[24] Yudin V.E., Svetlichnyi V.M., Shumakov A.N., Letenko D.G., Feldman A.Y., Marom G. (2005). The nucleating effect of carbon nanotubes on crystallinity in R-BAPB-type thermoplastic polyimide. Macromol. Rapid Commun..

[25] Yudin V.E., Feldman A.Y., Svetlichnyi V.M., Shumakov A.N., Marom G. (2007). Crystallization of R-BAPB type polyimide modified by carbon nano-particles. Compos. Sci. Technol..

[26] Ning N., Fu S., Zhang W., Chen F., Wang K., Deng H., Zhang Q., Fu Q. (2012). Realizing the enhancement of interfacial interaction in semicrystalline polymer/filler composites via interfacial crystallization. Prog. Polym. Sci..

[27] Batistakis C., Lyulin A.V., Michels M.A.J. (2012). Slowing down versus acceleration in the dynamics of confined polymer films. Macromolecules.

[28] Solar M., Paul W. (2015). Dielectric α-relaxation of 1,4-polybutadiene confined between graphite walls. Eur. Phys. J. E.

[29] Velasco-Santos C., Martinez-Hernandez A.L., Castano V.M. (2005). Carbon nanotube-polymer nanocomposites: The role of interfaces. Compos. Interfaces.

[30] Rahmat M., Hubert P. (2011). Interactions in nanocomposites: A review. Compos. Sci. Technol..

[31] Andrews R., Weisenberger M.C. (2004). Carbon nanotube polymer composites. Curr. Opin. Solid State Mater. Sci..

[32] Coleman J.N., Khan U., Blau W.J., Gun’ko Y.K. (2006). Small but strong: A review of the mechanical properties of carbon nanotube-polymer composites. Carbon.

[33] Mark J.E. (2007). Physical Properties of Polymers Handbook.

[34] Green M.J., Behabtu N., Pasquali M., Adams W.W. (2009). Nanotubes as polymers. Polymer.

[35] Baskaran D., Mays J.W., Bratcher M.S. (2005). Noncovalent and nonspecific molecular interactions of polymers with multiwalled carbon nanotubes. Chem. Mater..

[36] Tallury S.S., Pasquinelli M.A. (2010). Molecular dynamics simulations of flexible polymer chains wrapping single-walled carbon nanotubes. J. Phys. Chem. B.

[37] Steuerman D.W., Star A., Narizzano R., Choi H., Ries R.S., Nicolini C., Stoddart J.F., Heath J.R. (2002). Interactions between conjugated polymers and single-walled carbon nanotubes. J. Phys. Chem. B.

[38] Li H., Yan S. (2011). Surface-induced polymer crystallization and the resultant structures and morphologies. Macromolecules.

[39] Azimi M., Mirjavadi S.S., Hamouda A.M.S., Makki H. (2017). Heterogeneities in polymer structural and dynamic properties in graphene and graphene oxide nanocomposites: Molecular dynamics simulations. Macromol. Theory Simul..

[40] Yang M., Koutsos V., Zaiser M. (2005). Interactions between polymers and carbon nanotubes: A molecular dynamics study. J. Phys. Chem. B.

[41] Minoia A., Chen L., Beljonne D., Lazzaroni R. (2012). Molecular modeling study of the structure and stability of polymer/carbon nanotube interfaces. Polymer.

[42] Jiang Q., Tallury S.S., Qiu Y., Pasquinelli M.A. (2014). Molecular dynamics simulations of the effect of the volume fraction on unidirectional polyimide-carbon nanotube nanocomposites. Carbon.

[43] Liu W., Yang C.-L., Zhu Y.-T., Wang M. (2008). Interactions between single-walled carbon nanotubes and polyethylene/polypropylene/polystyrene/poly(phenylacetylene)/poly(*p*-phenylenevinylene) considering repeat unit arrangements and conformations: A molecular dynamics simulation study. J. Phys. Chem. C.

[44] Asadinezhad A., Kelich P. (2017). Effects of carbon nanofiller characteristics on ptt chain conformation and dynamics: A computational study. Appl. Surf. Sci..

[45] Yang H., Chen Y., Liu Y., Cai W.S., Li Z.S. (2007). Molecular dynamics simulation of polyethylene on single wall carbon nanotube. J. Chem. Phys..

[46] Yang J.-S., Yang C.-L., Wang M.-S., Chen B.-D., Ma X.-G. (2011). Crystallization of alkane melts induced by carbon nanotubes and graphene nanosheets: A molecular dynamics simulation study. Phys. Chem. Chem. Phys..

[47] Liaw D.-J., Wang K.-L., Huang Y.-C., Lee K.-R., Lai J.-Y., Ha C.-S. (2012). Advanced polyimide materials: Syntheses, physical properties and applications. Prog. Polym. Sci..

[48] Bessonov M.I., Koton M.M., Kudryavtsev V.V., Laius L.A. (1987). Polyimides: Thermally Stable Polymers.

[49] Yudin V.E., Svetlichnyi V.M., Gubanova G.N., Didenko A.L., Sukhanova T.E., Kudryavtsev V.V., Ratner S., Marom G. (2002). Semicrystalline polyimide matrices for composites: Crystallization and properties. J. Appl. Polym. Sci..

[50] Larin S.V., Falkovich S.G., Nazarychev V.M., Gurtovenko A.A., Lyulin A.V., Lyulin S.V. (2014). Molecular-dynamics simulation of polyimide matrix pre-crystallization near the surface of a single-walled carbon nanotube. RSC Adv..

[51] Larin S.V., Glova A.D., Serebryakov E.B., Nazarychev V.M., Kenny J.M., Lyulin S.V. (2015). Influence of the carbon nanotube surface modification on the microstructure of thermoplastic binders. RSC Adv..

[52] Falkovich S.G., Nazarychev V.M., Larin S.V., Kenny J.M., Lyulin S.V. (2016). Mechanical properties of a polymer at the interface structurally ordered by graphene. J. Phys. Chem. C.

[53] Falkovich S.G., Larin S.V., Lyulin A.V., Yudin V.E., Kenny J.M., Lyulin S.V. (2014). Influence of the carbon nanofiller surface curvature on the initiation of crystallization in thermoplastic polymers. RSC Adv..

[54] Lyulin S.V., Larin S.V., Gurtovenko A.A., Lukasheva N.V., Yudin V.E., Svetlichnyi V.M., Lyulin A.V. (2012). Effect of the SO_2_ group in the diamine fragment of polyimides on their structural, thermophysical, and mechanical properties. Polym. Sci. Ser. A.

[55] Lyulin S.V., Gurtovenko A.A., Larin S.V., Nazarychev V.M., Lyulin A.V. (2013). Microsecond atomic-scale molecular dynamics simulations of polyimides. Macromolecules.

[56] Lyulin S.V., Larin S.V., Gurtovenko A.A., Nazarychev V.M., Falkovich S.G., Yudin V.E., Svetlichnyi V.M., Gofmana I.V., Lyulin A.V. (2014). Thermal properties of bulk polyimides: Insights from computer modeling versus experiment. Soft Matter.

[57] Falkovich S.G., Lyulin S.V., Nazarychev V.M., Larin S.V., Gurtovenko A.A., Lukasheva N.V., Lyulin A.V. (2014). Influence of the electrostatic interactions on thermophysical properties of polyimides: Molecular-dynamics simulations. J. Polym. Sci. B.

[58] Nazarychev V.M., Larin S.V., Yakimansky A.V., Lukasheva N.V., Gurtovenko A.A., Gofman I.V., Yudin V.E., Svetlichnyi V.M., Kenny J.M., Lyulin S.V. (2015). Parameterization of electrostatic interactions for molecular dynamics simulations of heterocyclic polymers. J. Polym. Sci. B.

[59] Nazarychev V.M., Lyulin A.V., Larin S.V., Gurtovenko A.A., Kenny J.M., Lyulin S.V. (2016). Molecular Dynamics Simulations of Uniaxial Deformation of Thermoplastic Polyimides. Soft Matter.

[60] Nazarychev V.M., Lyulin A.V., Larin S.V., Gofman I.V., Kenny J.M., Lyulin S.V. (2016). Correlation between the high-temperature local mobility of heterocyclic polyimides and their mechanical properties. Macromolecules.

[61] Lyulin S.V., Larin S.V., Nazarychev V.M., Fal’kovich S.G., Kenny J.M. (2016). Multiscale computer simulation of polymer nanocomposites based on thermoplastics. Polym. Sci. Ser. C.

[62] Nazarychev V.M., Larin S.V., Lukasheva N.V., Glova A.D., Lyulin S.V. (2013). Evaluation of the characteristic equilibration times of bulk polyimides via full-atomic computer simulation. Polym. Sci. Ser. A.

[63] Hegde M., Lafont U., Norder B., Picken S.J., Samulski E.T., Rubinstein M., Dingemans T. (2013). SWCNT Induced crystallization in an amorphous all-aromatic poly(ether imide). Macromolecules.

[64] Hegde M., Lafont U., Norder B., Samulski E.T., Rubinstein M., Dingemans T.J. (2014). SWCNT Induced crystallization in amorphous and semi-crystalline poly(etherimide)s: Morphology and thermo-mechanical properties. Polymer.

[65] Van Der Spoel D., Lindahl E., Hess B., Groenhof G., Mark A.E., Berendsen H.J.C. (2005). GROMACS: Fast, flexible, and free. J. Comput. Chem..

[66] Hess B., Kutzner C., van der Spoel D., Lindahl E. (2008). GROMACS 4: Algorithms for highly efficient, load-balanced, and scalable molecular simulation. J. Chem. Theory Comput..

[67] Oostenbrink C., Villa A., Mark A.E., Van Gunsteren W.F. (2004). A biomolecular force field based on the free enthalpy of hydration and solvation: The GROMOS force-field parameter sets 53A5 and 53A6. J. Comput. Chem..

[68] Darden T., York D., Pedersen L. (1993). Particle mesh Ewald: An *N*·log(*N*) method for Ewald sums in large systems. J. Chem. Phys..

[69] Karatrantos A., Composto R.J., Winey K.I., Clarke N. (2011). Structure and conformations of Polymer/SWCNT nanocomposites. Macromolecules.

[70] Chakraborty S., Roy S. (2012). Structural, dynamical, and thermodynamical properties of carbon nanotube polycarbonate composites: A molecular dynamics study. J. Phys. Chem. B.

[71] Anwar M., Turci F., Schilling T. (2012). Crystallization mechanism in melts of short n-alkane chains. J. Chem. Phys..

[72] Anwar M., Berryman J.T., Schilling T. (2014). Crystal nucleation mechanism in melts of short polymer chains under quiescent conditions and under shear flow. J. Chem. Phys..

[73] Šponer J., Jurečka P., Marchan I., Luque F.J., Orozco M., Hobza P. (2006). Nature of base stacking: Reference quantum-chemical stacking energiesin ten unique B-DNA base-pair steps. Chem. Eur. J..

[74] Oostenbrink C., Soares T.A., Van Der Vegt N.F.A., Van Gunsteren W.F. (2005). Validation of the 53A6 GROMOS force field. Eur. Biophys. J..

[75] Weeks J.D., Chandler D., Andersen H.C. (1971). Role of Repulsive forces in determining the equilibrium structure of simple liquids. J. Chem. Phys..

[76] Chen J., Liu H., Weimer W.A., Halls M.D., Waldeck D.H., Walker G.C. (2002). Noncovalent engineering of carbon nanotube surfaces by rigid, functional conjugated polymers. J. Am. Chem. Soc..

[77] Chen R.J., Zhang Y., Wang D., Dai H. (2001). Noncovalent sidewall functionalization of single-walled carbon nanotubes for protein immobilization. J. Am. Chem. Soc..

