# Supplementary material for: Atomistic Molecular Dynamics Simulations of the Initial Crystallization Stage in an SWCNT-Polyetherimide Nanocomposite
Source: Polymers (Basel). 2017 Oct 24;9(10):548. doi: 10.3390/polym9100548 (PMC6418835; doi:10.3390/polym9100548)
Supplement: Supplementary file 1 [file polymers-09-00548-s001.pdf]

# *Supplementary Materials for*

## Atomistic Molecular Dynamics Simulations of the Initial Crystallization Stage in an SWCNT-Polyetherimide Nanocomposite

Victor M. Nazarychev,[1] Sergey V. Larin,[1] Alexey V. Lyulin,[2] Theo Dingemans,[3] Jose M. Kenny,[1,4] Sergey V. Lyulin[1,*]

[1]Institute of Macromolecular Compounds, Russian Academy of Sciences, Bol'shoi pr. 31 (V.O.), St. Petersburg, 199004, Russia

[2]Theory of Polymers and Soft Matter Group, Technische Universiteit Eindhoven, PO Box 513, 5600 MB Eindhoven, The Netherlands

[3]University of North Carolina at Chapel Hill, Department of Applied Physical Sciences, Murray Hall 1113, 121 South Road, NC 27599-3050, USA

[4]Materials Science and Technology Centre, University of Perugia, Loc. Pentima, 4, 05100 Terni, Italy

[*]Corresponding author. Tel: +7 812 3237407. E-mail: s.v.lyulin@gmail.com (Sergey V. Lyulin)

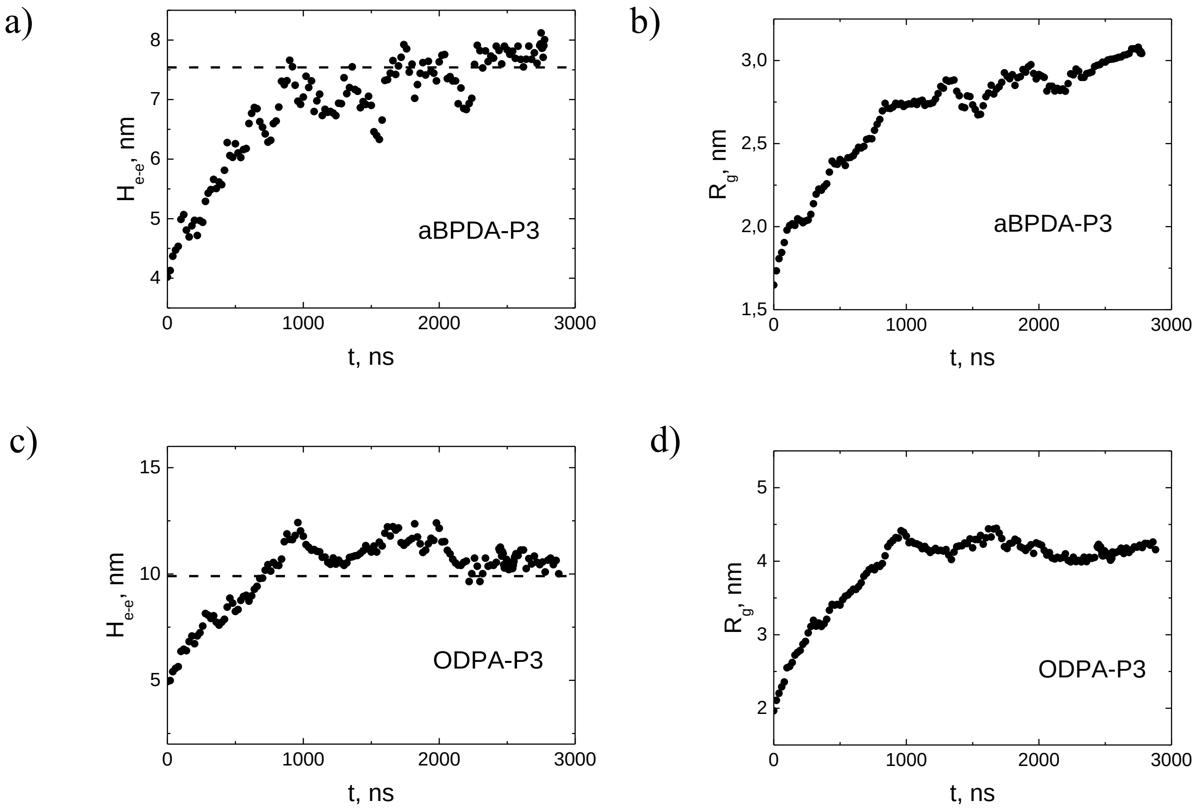


Figure S1. Time dependence of average end-to-end distance ($H_{e-e}$) and radius of gyration ($R_g$) for polyetherimides (PEIs) aBPDA-P3 (a, b) and ODPA-P3 (c, d). The dashed lines indicate the average end-to-end distances, calculated by virtual bond formalism based on the free rotation around the adjacent bonds.

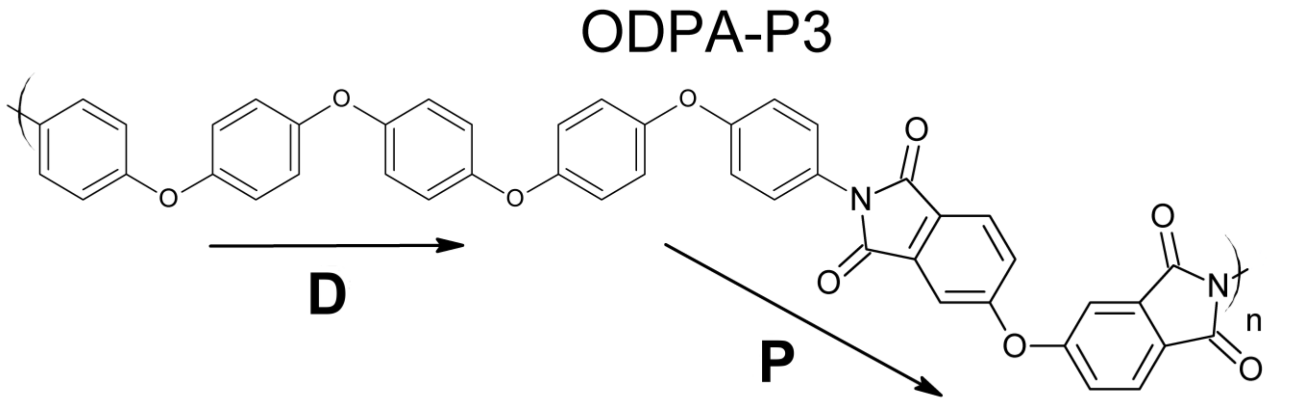


Figure S2. Chemical structure of repeat unit of the thermoplastic PEI ODPA-P3. Arrows mark the vectors **D** and **P** aligned along phenylene and phthalimide planar moieties of the polyimide chains under study which orientation to the nanotube axis was investigated in this paper. Phenylene rings in the moiety designated by **D** vector can rotate out of planar conformation, however such deviations from planar structure are small especially in the vicinity of CNT surface and phenylene rings are mostly coplanar.

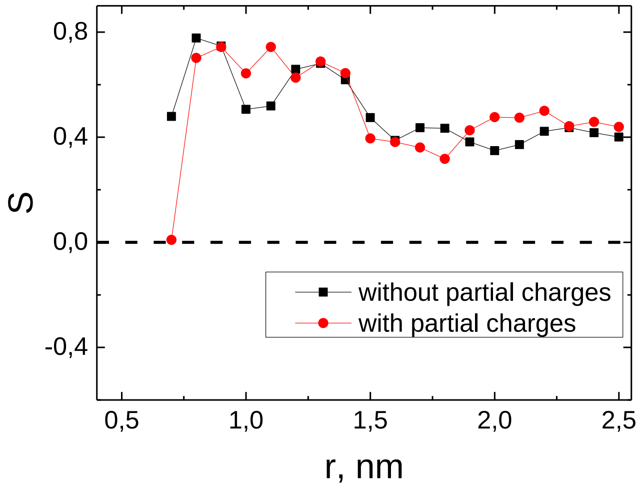


Figure S3. The order parameter $S(r)$ for the vector **P** in nanocomposites based on ODPA-P3 simulated with and without accounting for partial charges. The values of partial charges were calculated using the HF/6-31G* (Mulliken) method. The dashed line indicates zero value of order parameter.

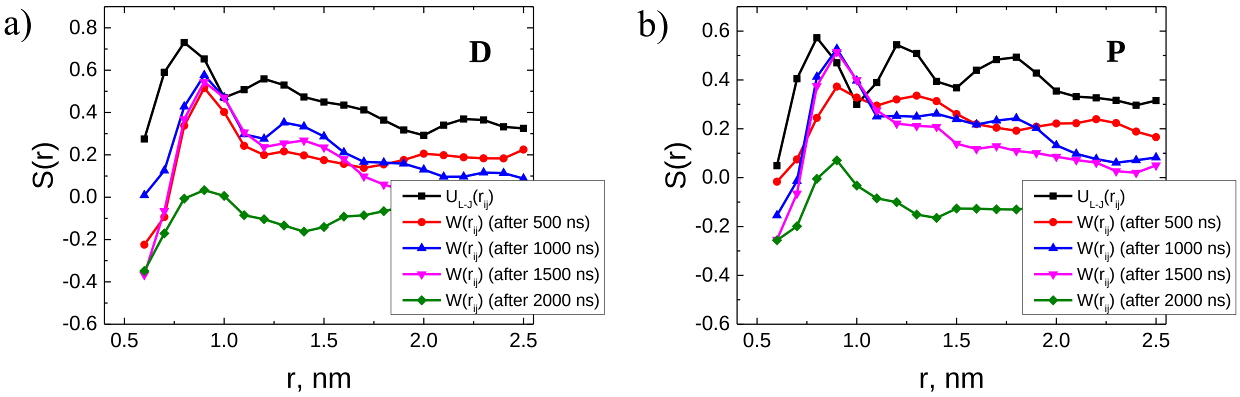


Figure S4. The order parameter $S(r)$ of the vectors a) **D** and b) **P** in nanocomposites based on ODPA-P3, calculated for the samples produced before and after switching between Lennard-Jones $U_{L-J}\left(r_{ij}\right)$ and Weeks-Chandler-Anderson $W\left(r_{ij}\right)$ potentials after different simulation times.

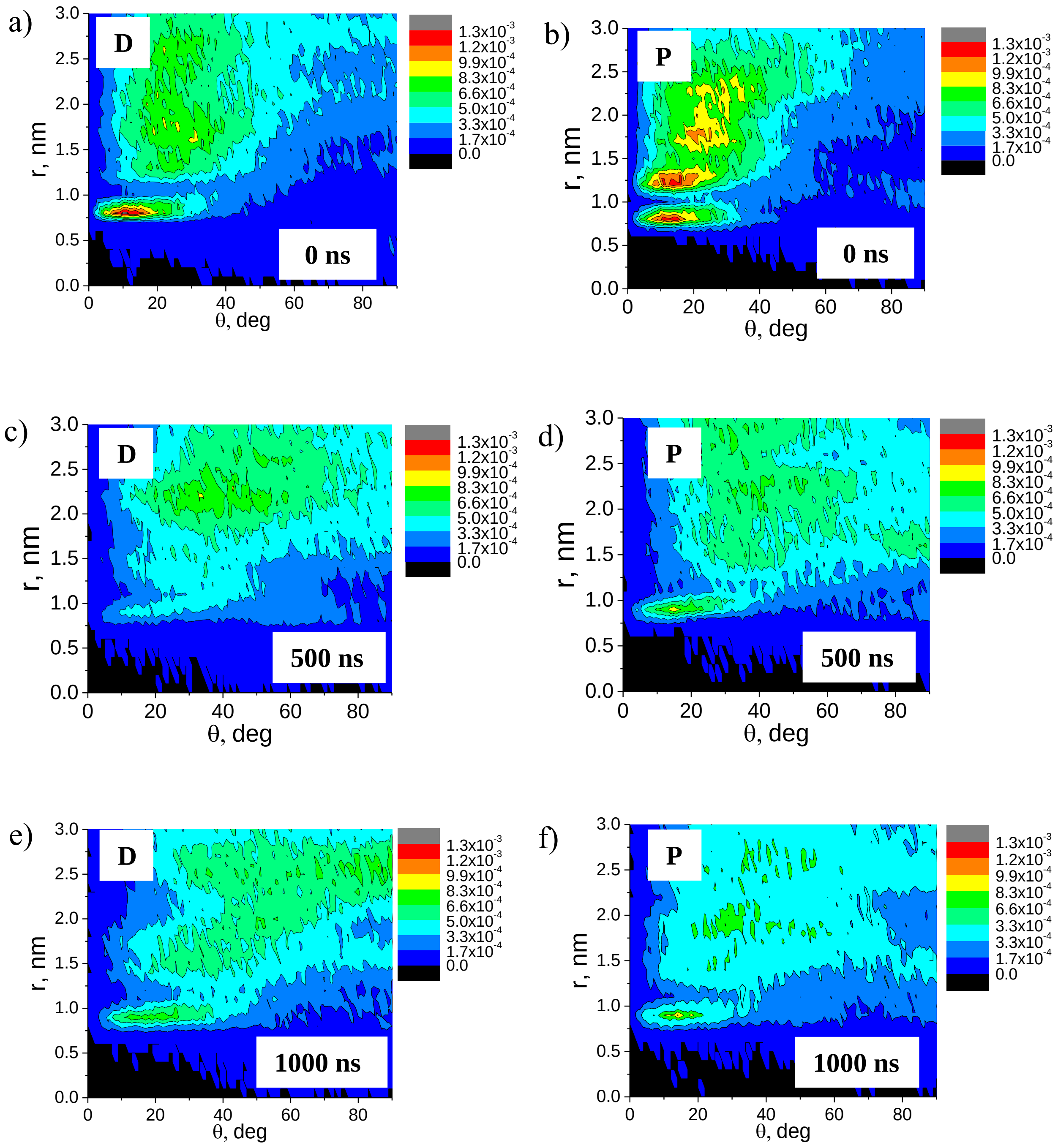

a)
D
0 ns
b)
P
0 ns
c)
D
500 ns
d)
P
500 ns
e)
D
1000 ns
f)
P
1000 ns
r, nm
θ, deg
1.3x10-3
1.2x10-3
9.9x10-4
8.3x10-4
6.6x10-4
5.0x10-4
3.3x10-4
1.7x10-4
0.0

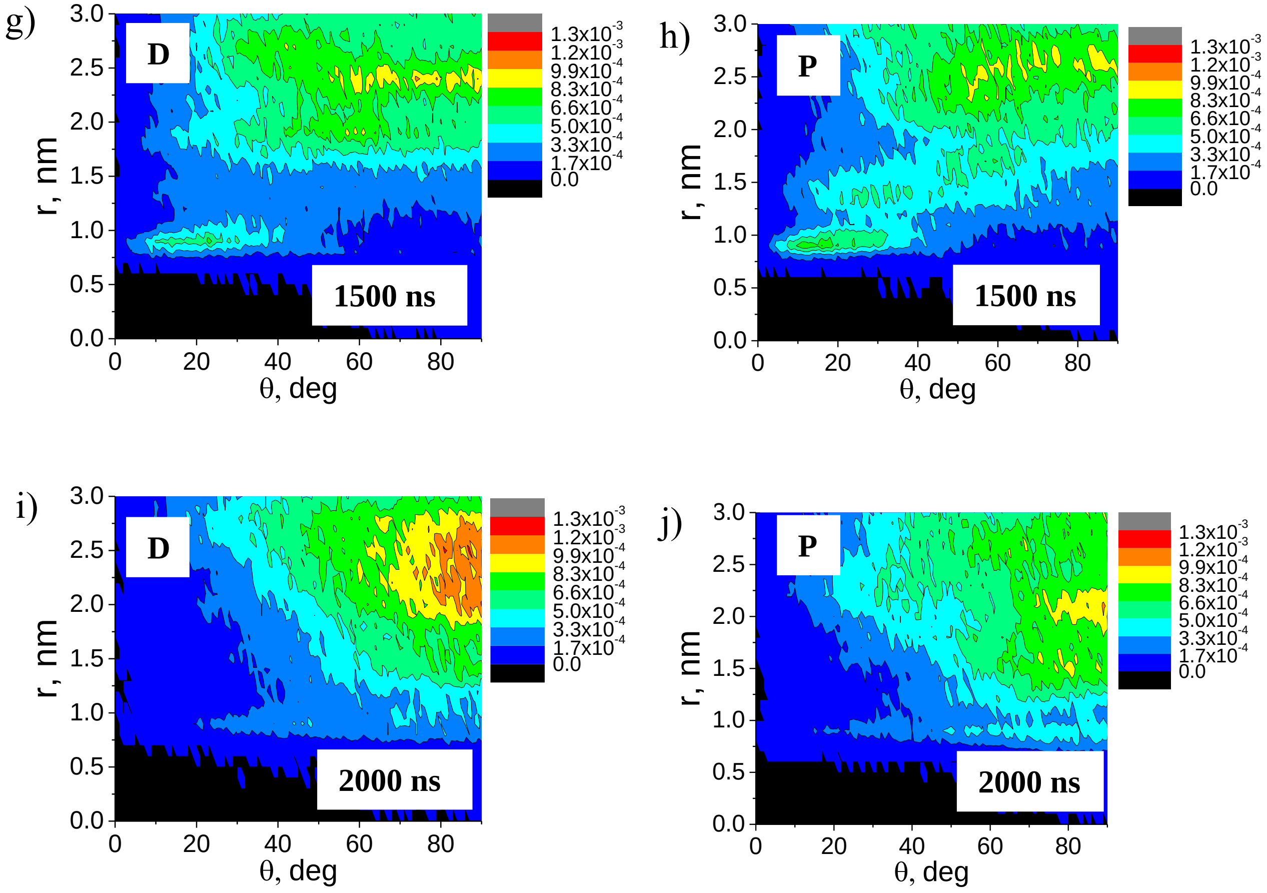


Figure S5. Distribution of orientation angles $\theta$ of vectors **D** (a, c, e, g, i) and **P** (b, d, f, h, j) in nanocomposites based on ODPA-P3 before (a, b) and after (c-j) switching between Lennard-Jones $U_{L-J}\left(r_{ij}\right)$ and Weeks-Chandler-Anderson $W\left(r_{ij}\right)$ potentials after different simulation times.

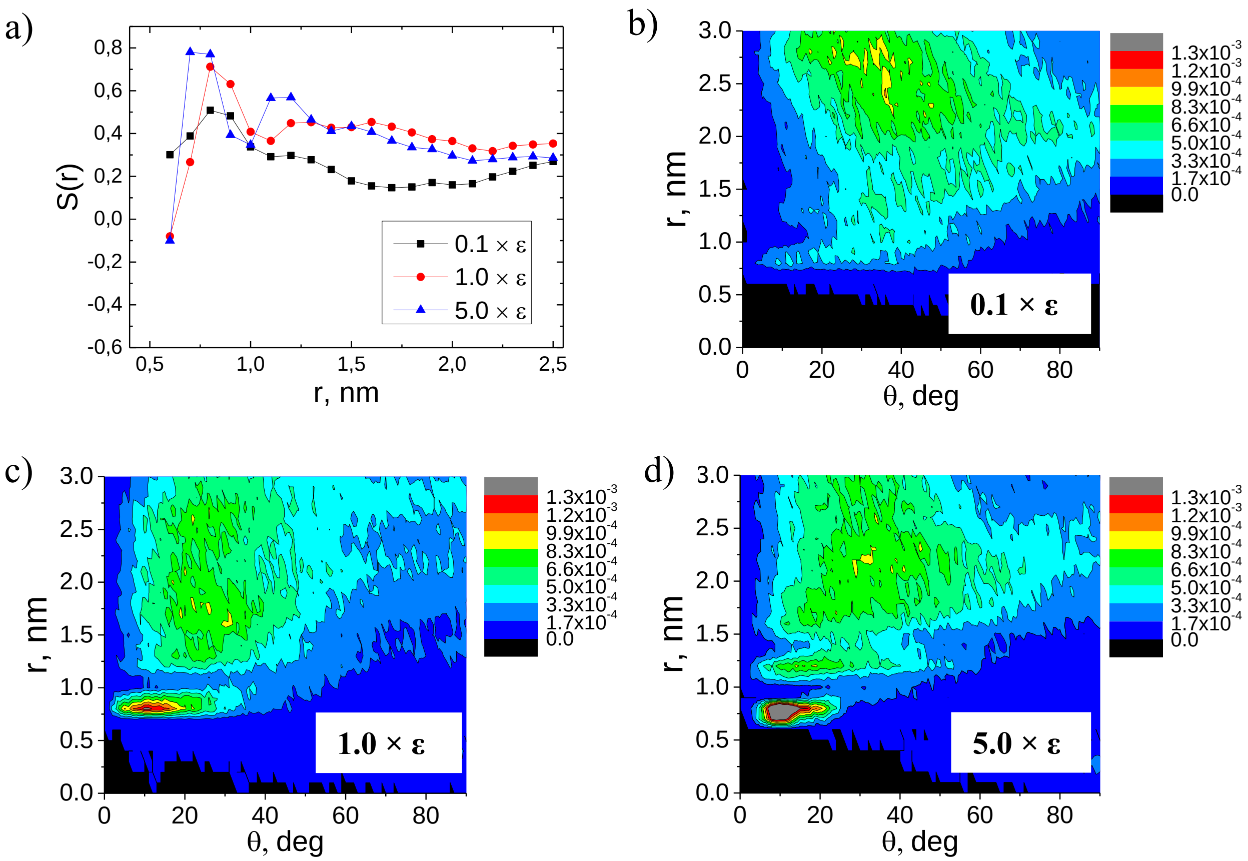


Figure S6. The order parameter $S(r)$ (a) and distribution of orientation angles $\theta$ of vectors **D** in nanocomposites based on ODPA-P3 at the increase in the potential well depth $\varepsilon$ by 0.1 (b), 1 (c), and 5 (d) times as compared to the initial parameters of the Gromos53a5 force field after 1 μs simulation time.
